# The phylogeography of trypanosomes from South American alligatorids and African crocodilids is consistent with the geological history of South American river basins and the transoceanic dispersal of *Crocodylus* at the Miocene

**DOI:** 10.1186/1756-3305-6-313

**Published:** 2013-10-29

**Authors:** Bruno R Fermino, Laerte B Viola, Fernando Paiva, Herakles A Garcia, Catia D de Paula, Robinson Botero-Arias, Carmen S A Takata, Marta Campaner, Patrick B Hamilton, Erney P Camargo, Marta MG Teixeira

**Affiliations:** 1 Departamento de Parasitologia, Instituto de Ciências Biomédicas, Universidade de São Paulo, São Paulo, SP 05508-900, Brazil; 2 Departamento de Parasitologia Veterinária, Universidade Federal do Mato Grosso do Sul, Campo Grande, Mato Grosso do Sul, Brazil; 3 Departamento de Patología Veterinaria, Faculdad de Ciencias Veterinarias, Universidad Central de Venezuela, Maracay, Aragua, Venezuela; 4 Faculdade de Agronomia e Medicina Veterinária, Universidade de Brasília, Brasília, DF, Brazil; 5 Caiman Research in Conservation and Management Program, Mamirauá Institute for Sustainable Development, Tefé, Amazonas, Brazil; 6 Biosciences, College of Life and Environmental Sciences, University of Exeter, Exeter, UK

**Keywords:** Crocodilian, *Trypanosoma* evolution, Historical biogeography, Host-switching, Phylogeography, Transoceanic dispersion, Disjunct distribution, South American river basins

## Abstract

**Background:**

Little is known about the diversity, phylogenetic relationships, and biogeography of trypanosomes infecting non-mammalian hosts. In this study, we investigated the influence of host species and biogeography on shaping the genetic diversity, phylogenetic relationship, and distribution of trypanosomes from South American alligatorids and African crocodilids.

**Methods:**

Small Subunit rRNA (SSU rRNA) and glycosomal Glyceraldehyde Phosphate Dehydrogenase (gGAPDH) genes were employed for phylogenetic inferences. Trypanosomes from crocodilians were obtained by haemoculturing. Growth behaviour, morphology, and ultrastructural features complement the molecular description of two new species strongly supported by phylogenetic analyses.

**Results:**

The inferred phylogenies disclosed a strongly supported crocodilian-restricted clade comprising three subclades. The subclade *T. grayi* comprised the African *Trypanosoma grayi* from *Crocodylus niloticus* and tsetse flies. The subclade *T. ralphi* comprised alligatorid trypanosomes represented by *Trypanosoma ralphi* n. sp*.* from *Melanosuchus niger, Caiman crocodilus* and *Caiman yacare* from Brazilian river basins. *T. grayi* and *T. ralphi* were sister subclades. The basal subclade *T. terena* comprised alligatorid trypanosomes represented by *Trypanosoma terena* n. sp. from *Ca. yacare* sharing hosts and basins with the distantly genetic related *T. ralphi.* This subclade also included the trypanosome from *Ca. crocodilus* from the Orinoco basin in Venezuela and, unexpectedly, a trypanosome from the African crocodilian *Osteolaemus tetraspis*.

**Conclusion:**

The close relationship between South American and African trypanosomes is consistent with paleontological evidence of recent transoceanic dispersal of *Crocodylus* at the Miocene/Pliocene boundaries (4–5 mya), and host-switching of trypanosomes throughout the geological configuration of South American hydrographical basins shaping the evolutionary histories of the crocodilians and their trypanosomes.

## Background

There is increasing evidence that the evolutionary histories of hosts and parasites are associated, and consequently, that parasites can serve as proxies to understand host evolutionary history and vice versa. Comparative phylogeography and biogeography of hosts and obligate parasites can reveal the impressions left on contemporary species by evolutionary processes such as co-evolution, dispersion and colonisation [1-7].

The genus *Trypanosoma* (Kinetoplastea, Trypanosomatidae) comprises worldwide parasites of all vertebrate classes transmitted by hematophagous vectors, such as insects, ticks and leeches [8,9]. Trypanosomes have been described in many lizards, snakes and crocodilians [10]. The evolutionary histories of trypanosomes from mammals of distinct orders and from other vertebrate classes only recently began to be addressed [11-19]. Trypanosomes parasitizing alligatorids have been reported in *Ca. crocodilus* and *Ca. yacare* from Brazilian Amazonia and Pantanal, respectively [20,21]. In Africa, trypanosomes of *Crocodylus niloticus, Mecistops cataphractus* and *Osteolaemus tetraspis* were all named *T. grayi*[22,23], a species that has been commonly described in its vector tsetse flies [24-27]. The transmission of *T. grayi* to crocodiles occurs by oral contamination with tsetse faeces or the ingestion of crushed flies [23]. The South American vectors of crocodilian trypanosomes are unknown. The first trypanosomes from alligatorids established as continuous cultures were from the Brazilian *Ca. yacare.* Nine isolates were molecularly characterised and assigned to two genotypes, Cay01 and Cay02, distinct from *T. grayi* from the African *C. niloticus*. However, previous phylogenetic analysis restricted to a few crocodilian trypanosomes hampered any attempt to evaluate species richness, and to infer biogeographical scenarios underlining the evolutionary history of crocodilian trypanosomes [8,9,12,21].

In an evolutionary story that spans more than 200 million years, from the late Triassic to present, a very large diversity of crocodilians occupied worldwide habitats, but only a limited number of species survived the long and intricate Crocodylia evolutionary history [28-33]. The Crocodylia comprises species of Crocodylidae, inhabiting all continents, and of Alligatoridae, which are exclusive to the Western hemisphere (except for the Chinese alligator). The Alligatoridae and Crocodylidae diverged in the late Cretaceous (~90 mya) [28,32]. *M. niger* occurs in the Amazon basin; *Ca. crocodilus* is the most widespread species in the Latin America; and *Ca. yacare* occurs mainly in the Paraguay/Paraná basin [34]. The current limited distribution and small number of *Crocodylus* species in South America (*C. intermedius* in the Orinoco basin and *C. acutus* from northwest Colombia/Venezuela) contrast with great diversity and wide distribution of fossils at the Miocene (9–5 mya) [35,36]. *C. niloticus* is the most widespread crocodilian species in Africa, and more closely related to South American than to Asian species of *Crocodylus*, whereas the African *Osteolaemus tetraspis* together with *Mecistops cataphractus* constitute the sister clade of *Crocodylus*[29-32].

In this study, we characterized trypanosomes from the South American *Ca. crocodilus, M. niger* and *Ca. yacare* and the African *C. niloticus* and *O. tetraspis* aiming: a) to evaluate the relative forces of host species and geography in shaping the genetic diversity, emergence of new species, phylogenetic relationships and distribution of crocodilian trypanosomes; b) to hypothesise paleontological and biogeographical scenarios consistent with both trypanosome and crocodilian evolutionary histories.

## Methods

### Collection sites, handling of the crocodilians, and culture of trypanosomes

**Figure 1:**
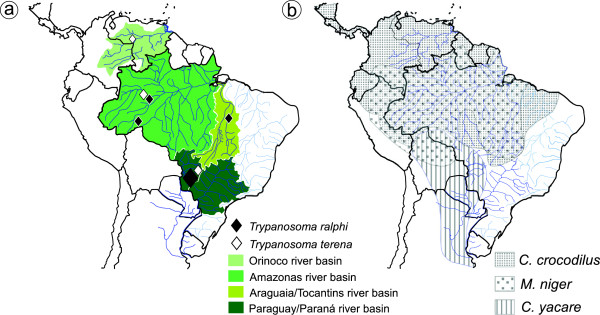
**Geographical distribution of *Trypanosoma ralphi, Trypanosoma terena *** **and allied trypanosomes (a), and the alligatorid host species (b) throughout the South American river basins (a,b).**

From 2002 to 2012 we captured crocodilians in the Paraguay-Paraná (PP), Amazonian (AM), Araguaia-Tocantins (AT) and Orinoco (OR) basins. *Caiman yacare* (19 animals) was captured in the Miranda River in the PP basin (S20^o^14´ W55^o^28´). *Ca. crocodilus* (4 animals) was captured in Brazil in the Purus River (S7^o^15´W64^o^47´) of the AM basin, and in the Araguaia River (S7^o^32´ W49^o^22´) of the AT basin (2 animals). Two *Ca. crocodilus* were captured in the Capanaparo River (Santos Luzardo National Park, Apure, Venezuela) (N6^o^83´ W67^o^69´) of the OR basin. *M. niger* was captured in Brazil, at the Purus River (two animals), and the Solimões River at the Mamirauá Reserve (5 animals) (S3^o^35´W64^o^72´), both in the AM basins. One specimen of *O. tetraspis* was captured in the National Park of Lagoas de Cufada in Guinea Bissau (S11^o^60´ W15^o^04´) (Figure 1, Table 1).

**Table 1:** Trypanosome isolates from South American and African crocodilians, host and geographic origin, and identification using sequences from SSU rRNA and gGAPDH genes

***Trypanosoma *** **Isolate**	**Host species**	**Geographic origin Locality/State/country**	**River Basin**	**Date**	**Sequence: GenBank acession numbers**	
					**SSU rRNA**	**gGAPDH**
***T. terena *** **subclade**						
TCC^a^ 610	*Caiman yacare*	Miranda/MS/Brazil	PP	2002	EU5962522	EU596256
TCC 1611	*Caiman yacare*	Miranda/MS/ Brazil	PP	2005	KF546517	KF546503
BSC^b^ 28	*Caiman crocodilus*	Achaguas/Apure/Venezuela	OR	2005	KF546518	KF546504
BSC 50	*Melanosuchus niger*	Mamirauá/AM/ Brazil	AM	2010	KF546519	
BSC 53	*Melanosuchus niger*	Mamirauá/AM/ Brazil	AM	2010	KF546520	
BSC 27	*Osteolaemus tetraspis*	Quinara/Guinea Bissau		2012		KF546505
***T. ralphi *** **subclade**						
TCC 624	*Caiman yacare*	Miranda/MS/Brazil	PP	2002	EU596253	EU596257
TCC 625	*Caiman yacare*	Miranda/MS/Brazil	PP	2002	EU596259	KF546506
TCC 1092	*Caiman yacare*	Miranda/MS/Brazil	PP	2005	EU596254	EU596258
TCC 1100	*Caiman yacare*	Miranda/MS/Brazil	PP	2005	EU596260	
TCC 1101	*Caiman yacare*	Miranda/MS/Brazil	PP	2005	EU596261	KF546507
TCC 1102	*Caiman yacare*	Miranda/MS/Brazil	PP	2005	EU596262	KF546508
TCC 1119	*Caiman yacare*	Miranda/MS/Brazil	PP	2005	EU596263	KF546509
TCC 1120	*Caiman yacare*	Miranda/MS/Brazil	PP	2005	EU596255	KF546510
TCC 1829	*Caiman crocodilus*	Lábrea/AM/Brazil	AM	2009	KF546521	KF546511
TCC1838	*Melanosuchus niger*	Lábrea/AM/Brazil	AM	2009	KF546527	KF546512
TCC 1974	*Caiman yacare*	Miranda/MS/Brazil	PP	2009	KF546522	KF546513
TCC 2218	*Caiman crocodilus*	Araguaína/TO/Brazil	AT	2011	KF546523	KF546514
BSC 29	*Caiman yacare*	Miranda/MS/Brazil	PP	2011		KF546515
BSC 51	*Melanosuchus niger*	Mamirauá/AM/Brazil	AM	2010	KF546524	
BSC 56	*Melanosuchus niger*	Mamirauá/AM/Brazil	AM	2010	KF546525	
BSC 64	*Caiman crocodilus*	Araguaína/TO/Brazil	AT	2011		KF546516
***T. grayi *** **subclade**						
BAN 1^c^	*Glossina palpalis* tsetse fly	The Gambia		-	AJ620258	AJ620258
ANR 4 ^c^	*Glossina palpalis* tsetse fly	The Gambia		-	AJ620257	AJ62025
CroCamp1^c^	*Crocodylus niloticus*	Cameroon		-	KF546526	FM164795

a, TCC, Codes of cultures cryopreserved at the Trypanosomatid Culture Collection of the University of São Paulo; b, BSC, Blood Sample Collection; c, DNA samples. Samples analyses for the first time in this study are those obtained from 2005 to 2011; the Genbank accession numbers of DNA sequences determined in this study are KF546503-27.

Brazilian States: MS, Mato Grosso do Sul; AM, Amazonas; TO, Tocantins. River Basins: PP, Paraguay/Paraná; OR, Orinoco; AM, Amazon; AT, Araguaya/Tocantins.

All procedures were performed according to the recommendations of IBAMA (the Brazilian Institute for the Environment and Renewable Natural Resources; Permit Number: 10080–2). The work in Venezuela and Guinea Bissau was carried out with the permission of local institutions. Animal handling was performed in strict accordance with good animal practice, as determined by the protocols (108/2003 and 021/2012) approved by the Committee on the Ethics of Animal Experimentation of the University of São Paulo, Institute of Biomedical Sciences.

After immobilisation, the animals were bled by tail puncture using sodium citrate as an anticoagulant. The distribution of the crocodilian species and locals within each South American hydrographic basin where the animals were captured are shown in the Figure 1a, Table 1. We examined crocodilian blood for trypanosomes under light microscopy in Giemsa-stained smears on glass slides. Blood samples (~ 1.0 ml) were immediately inoculated into tubes containing BAB-LIT medium, and the positive haemocultures were transferred to monolayers of Hi-5 feeder cells cultivated in TC100 medium as previously described [14,19,21]. All cultures were deposited in the Trypanosomatid Culture Collection of the University of São Paulo (TCC-USP) (Table 1).

### DNA amplification, sequencing and data analyses

DNA preparations from cultured trypanosomes were obtained using the classical phenol-chloroform method. The DNA preparations from blood samples were obtained from samples preserved in ethanol (v/v) stored in our Blood Sample Collections (BSC). DNA of *T. grayi* (CroCamp1) of *C. niloticus* from Cameroon, included in a previous study [12], was kindly provided by S. Helder, University of Yaoundé, Cameroon. PCR-amplification and sequencing of the V7V8 region of SSU rRNA (small subunit of rRNA) and glycosomal glyceraldehyde phosphate dehydrogenase (gGAPDH) genes were performed as described previously [37,38]. The sequences obtained were employed for the following phylogenetic analyses: a) V7V8 SSU rRNA sequences (880 ~bp) from the crocodilian trypanosomes; b) gGAPDH sequences (864 bp) from the crocodilian trypanosomes and species representing all major clades within *Trypanosoma* using non-trypanosome trypanosomatids as an outgroup; and c) concatenated V7V8 SSU rRNA and gGAPDH sequences (~3.3 kb) from the crocodilian trypanosomes and their closest related trypanosomes*.* The species included in the phylogenetic trees and their respective GenBank accession numbers are shown in in the phylogenetic trees and Table 1. The alignments were employed for Parsimony (V7V8 SSU rRNA), maximum likelihood (ML) and Bayesian inference (BI) analyses as previously described [15,19,21,37,38].

### Light, Scanning (SEM) and Transmission (TEM) Electron Microscopy

For light microscopy, cultured trypanosomes were fixed with methanol and stained with Giemsa. For Scanning (SEM) and Transmission (TEM) Electron Microscopy, trypanosomes from Hi-5 cultures were prepared and observed as previously described [14,19,37,38].

## Results

### Isolation in culture and growth behaviour

We collected blood from crocodilians captured in four South American river basins: PP, AM, AT and OR (Figure 1; Table 1) and characterized 5 new cultures: two from *Ca. yacare* (TCC1611, 1974), two from *Ca. Crocodilus* (TCC 1829, 2218) and one from *M. niger* (TCC 1838). We observed accentuated growth differences by culture comparison. The absence of Hi-5 cells markedly reduced the multiplication of the TCC 610 and 1611 isolates, whereas the remaining isolates easily adapted to grow on TC100 or LIT media without the feeder layer cells.

One primary culture (BSC28) was obtained from *Ca. crocodilus* from Venezuela (Table 1). This isolate and other Brazilian isolates failed to propagate beyond the first passage in culture. Some blood samples that tested positive for trypanosomes by microscopic investigation of fresh blood could not be cultured at all. To overcome this limiting factor, DNA isolated directly from crocodilian blood samples was used for PCR amplification and sequencing of the V7V8 SSU rRNA and/or gGAPDH sequences.

### Barcoding, species diversity and the phylogenetic relationships of crocodilian trypanosomes

**Figure 2:**
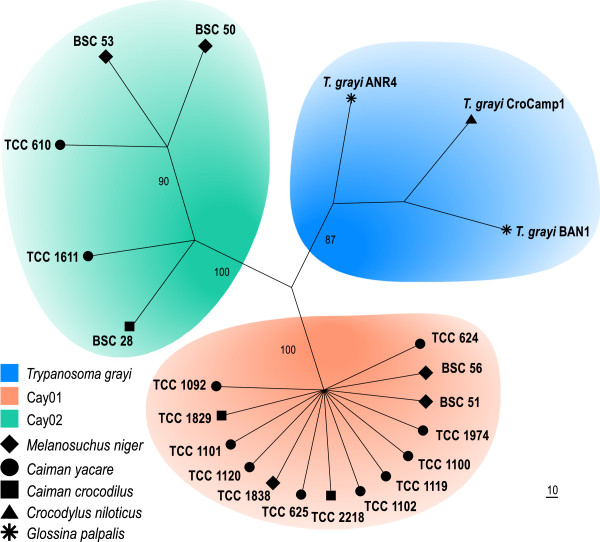
**Barcoding of crocodilian trypanosomes.** Dendrogram inferred using V7V8 SSU rRNA sequences (DNA barcodes) of 22 isolates from 5 species of crocodilians revealing three subclades of trypanosomes: *T. terena*, *T. ralphi* and *T. grayi*. The node numbers are bootstrap values derived from 100 replicates.

In previous studies, we demonstrated that barcoding using V7V8 SSU rRNA sequences is valuable for rapid species identification, and for revealing intra-specific diversity of trypanosomes in mammals [16-19] anurans [15], and snakes [13,14]. We previously employed this marker to characterise the genotypes Cay01 and Cay02 in *Ca. yacare*[21]. Here, we barcoded two new cultures from *Ca. yacare*, two from *Ca. crocodilus,* and one from *M. niger.* Barcoding the trypanosomes directly from crocodilian blood samples disclosed sequences that were identical to Cay01 or Cay02 genotypes (Table 1; Figure 2).

The SSU rRNA sequences obtained from the 5 new established cultures, the primary culture derived from the Venezuelan *Ca. crocodilus,* and 8 sequences obtained directly from the DNA from blood samples of *M. niger, Ca. yacare* and *Ca. crocodilus* and *O. tetraspis* were aligned with those previously determined [8,9,12,21] for 9 isolates from *Ca. yacare* and three isolates of *T. grayi,* including the trypanosome from *C. niloticus* from Cameroon barcoded in this study (Table 1). The inferred dendrogram corroborated the previous separation [21] of the alligatorid trypanosomes into the main genotypes Cay01 and Cay02, both distinct from *T. grayi* (Figure 2). The barcode analysis showed that Cay01 and Cay02 diverged ~2.5%, Cay02 and *T. grayi* also diverged ~2.5%, whereas a smaller divergence (1.3%) separated Cay01 from *T. grayi*.

**Figure 3:**
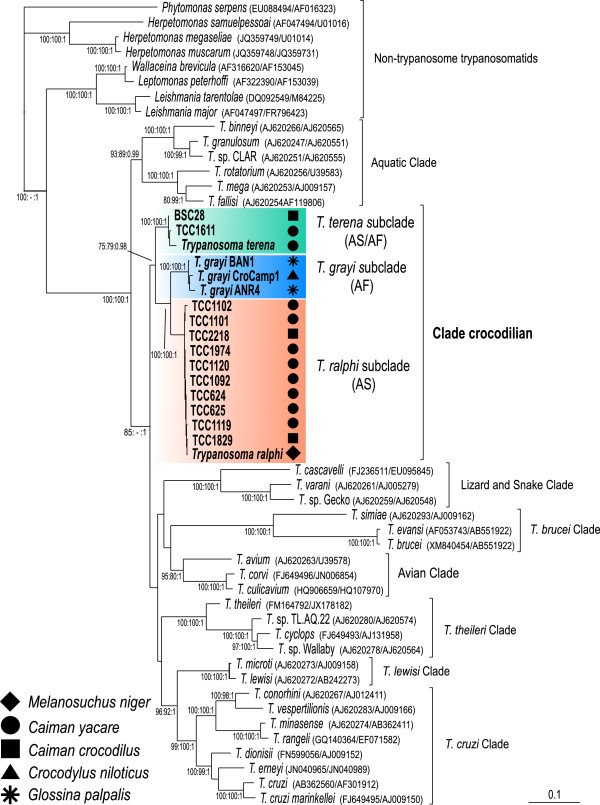
**Phylogenetic positioning of the crocodilian trypanosomes from South America and Africa.** Phylogenetic tree inferred by maximum likelihood (ML) of concatenated gGAPDH and V7V8 SSU rRNA sequences from 17 trypanosome isolates from crocodilians evidencing the Crocodilian clade and its three subclades *T. terena*, *T. ralphi* and *T. grayi.* The analyses include species representative of all major clades within the genus *Trypanosoma,* and trypanosomatids of other genera as outgroups (1.778 characters, – Ln = -17937.481223). Numbers at nodes are bootstrap support (P/ML) >50% or Bayesian posterior probability > 0.25, derived from 500 replicates. Codes within parenthesis are GenBank accession numbers.

**Figure 4:**
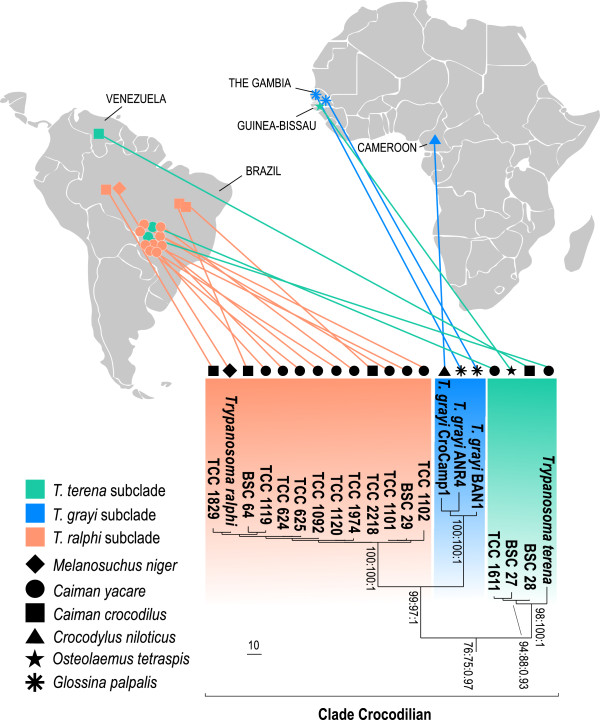
**Phylogeographical analysis of South American alligatorid and African crocodilid trypanosomes:** Phylogenetic relationships and geographical origin of trypanosomes from South American alligatorids, African crocodilids, and tsetse flies. ML phylogeny based on gGAPDH sequences (810 characters, –Ln = -10359.469307) from the trypanosomes nested into the Crocodilian clade (GenBank accession numbers are on Table 1), and trypanosomes from other hosts (GenBank accession numbers are within parenthesis on the tree). Numbers at nodes are bootstrap support >50% (P/ML) or Bayesian posterior probability > 0.25, derived from 500 replicates.

The gGAPDH gene (single copy gene) sequence can be more valuable than SSU rRNA genes (multiple copies) for the phylogenetic positioning of trypanosomatids [9,14,15,17,18,37,38]. Here, we showed that among the crocodilian trypanosomes, gGAPDH diverged more than SSU rRNA. The gGAPDH+V7V8 SSU rRNA (Figure 3) or gGAPDH (Figure 4) derived phylogenetic trees supported the monophyly of crocodilian trypanosomes resulting in a well-supported assemblage named the Crocodilian clade. This clade comprises three well-supported subclades and confirms the branching pattern inferred by the V7V8 SSU rRNA (Figure 1). The Crocodilian clade was positioned closer to the major assemblage of trypanosomes from mammals and from Archosaurian hosts, such as birds, lizards and snakes, than to the trypanosomes from fishes and anurans nested into the Aquatic clade (Figures 3, 4).

The results strongly support a crocodilian clade that consists exclusively of and comprises all trypanosomes from crocodilians, including isolates obtained from tsetse flies (Figures 3, 4). The positioning in the phylogenetic trees and gGAPDH sequence divergences, permitted classification of the Brazilian isolates into two new species, *Trypanosoma terena* n. sp. and *Trypanosoma ralphi* n. sp. The gGAPDH divergence between *T. ralphi* (the isolate TCC1838 from *M. niger* captured in the AM basin represents the type species) and *T. terena* (the isolate TCC610 from *C. yacare* captured in PP basin represents the type species) was 7.6%. The divergence separating *T. ralphi* and *T. grayi* was 7.0%, whereas the largest distances (9.5%) separated *T. terena* from *T. grayi.*

The phylogenetic analyses supported three subclades within the clade Crocodilian*.* The basal subclade comprised *T. terena* and four isolates from South America, one from *Ca. yacare,* one from *Ca. crocodilus* and two from *M. niger*, and also included the trypanosome from the African *O. tetrapsis*. The second subclade clustered together with *T. ralphi* from 16 Brazilian isolates, 10 from *Ca. yacare*, three from *Ca. crocodilus,* and three from *M. niger.* The third subclade was formed exclusively by African isolates, which grouped *T. grayi* of *C. niloticus* from Cameroon, and two tsetse isolates from The Gambia. *T. ralphi* and *T. grayi* are sister clades (Figures 3, 4). The most homogeneous subclade (< 0.5% gGAPDH divergences) was represented by *T. ralphi,* and South American isolates. The only exception within this clade was the TCC 1102 isolate, which diverged by ~1.0% from all isolates. Within the subclade *T. terena*, the isolate TCC 610 diverged by higher distances: 1.8% for the isolate from *O. tetrapsis,* 1.3% for the *Ca. crocodilus* isolate from OR, and 1.4% for the *C. yacare* isolate TCC1611 from PP. Considering that the divergences within the subclades are too small to justify the creation of new species, the isolates were designed as genotypes of the leading species of their respective subclades.

The African isolates from *C. niloticus* and *O. tetrapsis* were positioned into the two distant subclades *T. grayi* and *T. terena* (Figures 3, 4), and were separated by the largest gGAPDH divergences (9.6%)*.* The divergences separating the African trypanosomes were larger than those separating *T. grayi* from the South American trypanosomes nested into the subclade *T. ralphi* (6.3%). Divergences separating the isolates of *T. grayi* from *C. niloticus* (Cameroon) and tsetse flies (The Gambia) were 1.2% (isolate BAN1) and 1.7% (isolate ANR4), whereas the two tsetse isolates diverged by 1.9%. These findings underline the heterogeneity of the African crocodilian trypanosomes, all assigned to *T. grayi*, which probably comprise more than one species.

### Light microscopy and the development and differentiation of the new species of alligatorid trypanosomes

**Figure 5:**
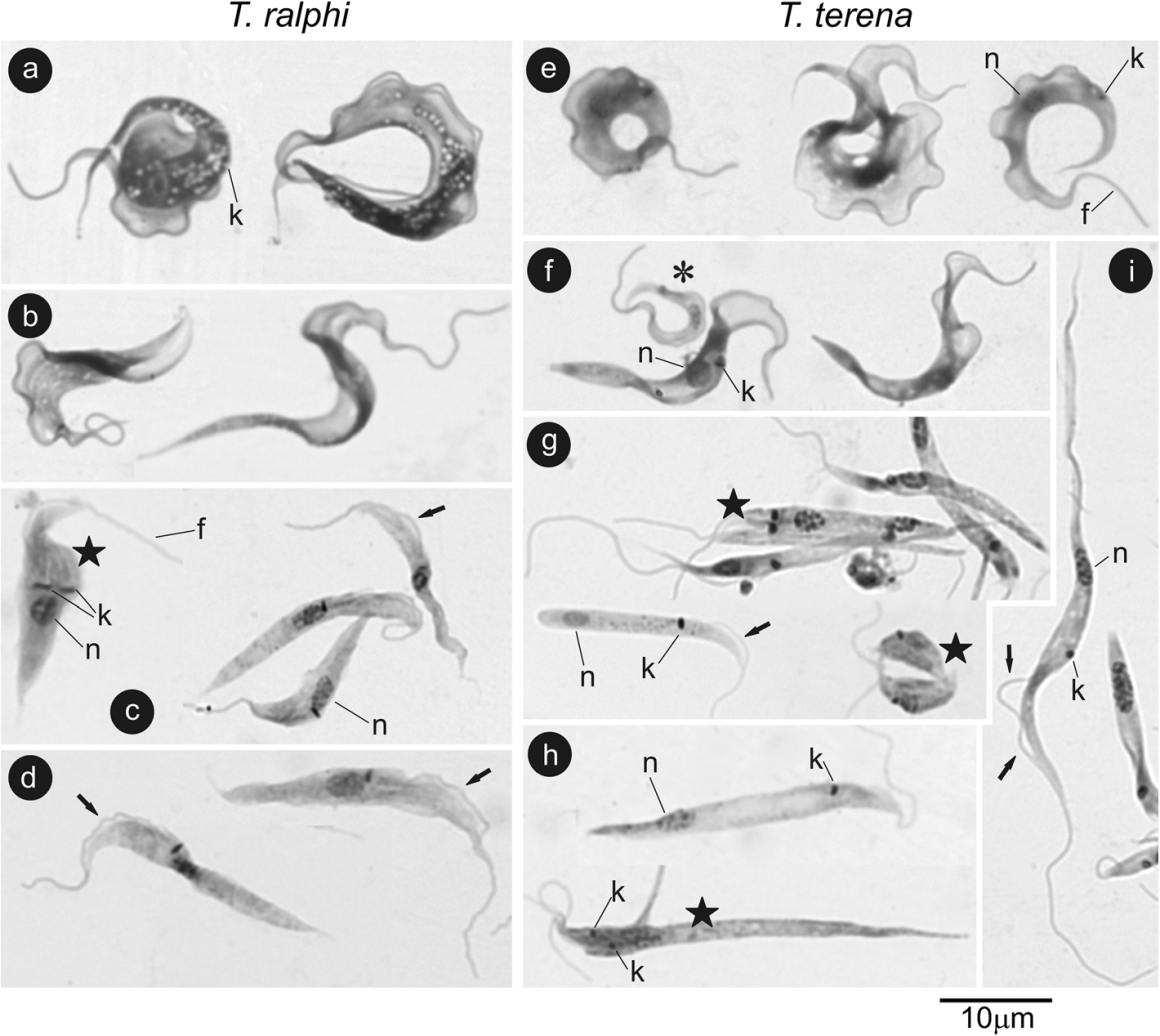
**Microphotographs (light microscopy) of Giemsa-staining culture forms of *Trypanosoma ralphi *(a-d) and *Trypanosoma terena *(e-i).** Cultures (~5 days) showing C- and roll-shaped large trypomastigotes with small kinetoplast and exuberant “undulant membrane” **(a,e)**, transient forms between trypo- and epimastigotes **(b,f)**, and small trypomastigotes (asterisk) (~7 days). Log-phase forms **(c,g)** showing rounded and long dividing forms (stars). The *T. ralphi* epimastigotes are pointed, exhibit a dilated anterior end **(c)**, and the kinetoplast is thin and adjacent to the nucleus **(c,d)**. The epimastigotes of *T. terena* are slender and the kinetoplast is rounded, and either very close or far from the nucleus **(g,h)**; wider forms predominated in stationary cultures **(h,i)**. Arrows indicate segments of the flagellar membrane detached from the cell body membrane. Nucleus (n), kinetoplast (k), flagellum (f).

An examination of the Giemsa-stained blood smears revealed a small number of C-shaped and roll-shaped (rounded forms with overlapping extremities) trypomastigotes in *Ca. yacare* blood samples as previously observed [21]. Blood flagellates were not observed in other crocodilian species. Hemocultures in TC100 using Hi-5 as feeder cells exhibited flagellates resembling blood trypomastigotes (Figure 5a,e). After ~7 days, the flagellates gradually differentiated to epimastigotes, and a few small trypomastigotes appeared in *T. terena* cultures (Figure 5b,f).

The flagellates from log-cultures ranged from small rounded forms to large epimastigotes. In *T. ralphi* (TCC 1838), epimastigotes varied in length and were generally wide forms with bodies measuring 17.5 to 32.5 μm long (average 23.26±4.48 μm) and 1.7 to 3.5 μm wide (average of 2.76±0.50 μm) showing a pointed posterior extremity, and dilated anterior end particularly in the stationary phase (Figure 5b,d). *T. terena* (TCC 610) epimastigotes were also pleomorphic in shape and size. Their bodies in log-phase ranged from 15 to 27.5 μm long (average of 22.2±3.7 μm), and 1.35 to 3.0 μm width (average of 2.06±0.44 μm) showing a rounded or pointed posterior extremity (Figure 5g). Long and slender epimastigotes ranging from ~30 to 40 μm long (average of 35.5±6.8 μm), and slim and twisted forms (Figure 5h,i) were common in the stationary cultures. Consistently, the epimastigotes of both species exhibited segments of the flagellar membrane that were detached from the cell body membrane before the emergence of free flagellum (Figure 5c,d,i). *T. ralphi* exhibited a thin kinetoplast adjacent to the central nucleus (Figure 5c,d), whereas the kinetoplasts in *T. terena* were rounded, compacted, and positioned either close or far from the nucleus (Figure 5g,h).

### Scanning (SEM) and transmission electron microscopy (TEM) of cultured trypanosomes

**Figure 6:**
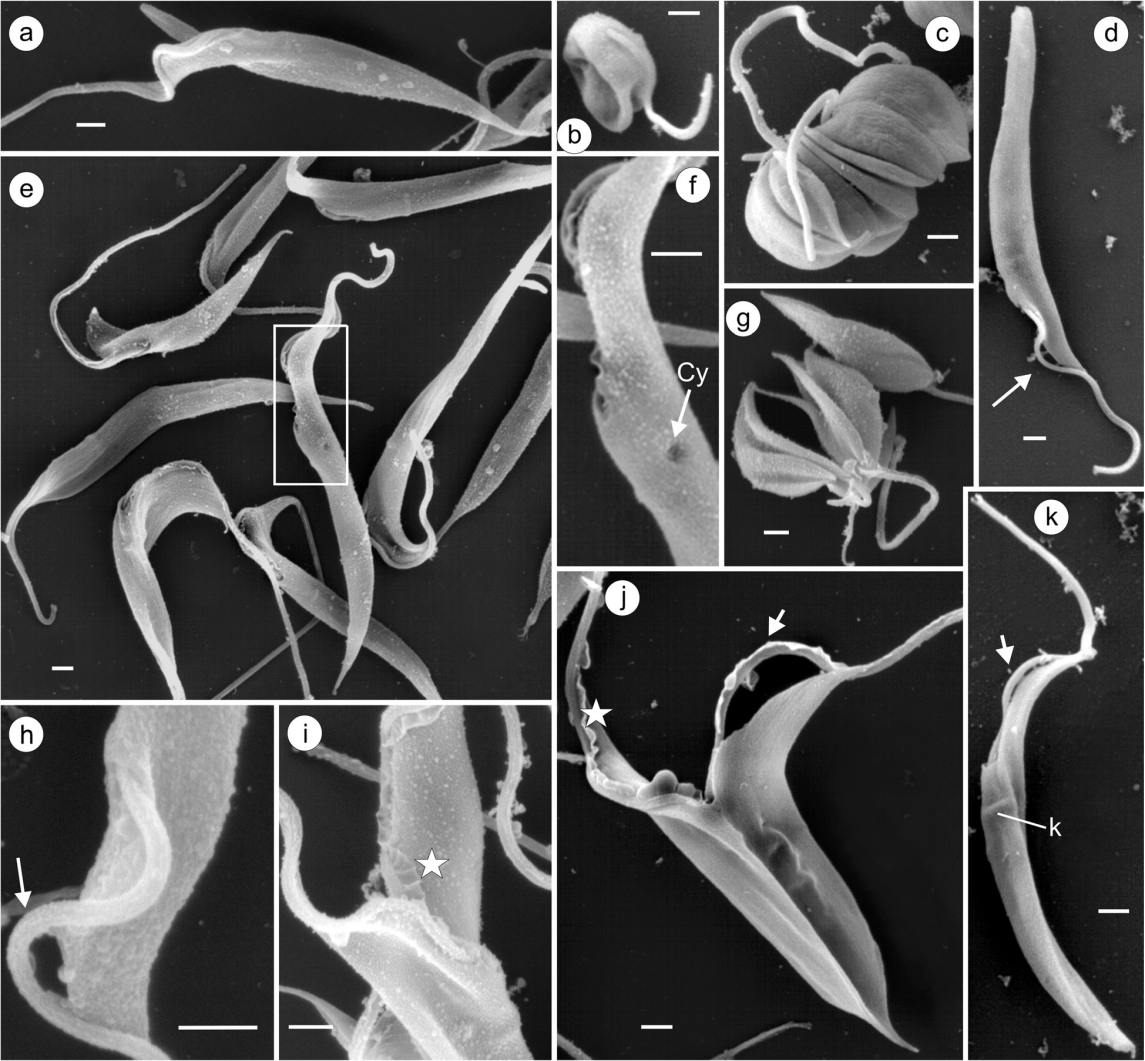
**Scanning electron microscopy of epimastigote forms of *Trypanosoma ralphi *(a,b,c,e,f,h,i) and *Trypanosoma terena *(d, g,j,k).** Log-phase epimastigotes of *T. ralphi* showing slender and pointed forms **(a,e)**, and of *T. terena***(d,k)** exhibiting rounded posterior extremity and a membrane protuberance corresponding to the kinetoplast **(k)**. Both species exhibited squash-like forms dividing by multiple fissions **(b,c,g)**, and long epimastigotes dividing by binary fission **(j)**. Higher magnification of the cytostome opening **(f)**, flagellar membrane detached from the cell body membrane **(d,h,j,k)**, and flagellar lamellae indicated by stars **(i,j)**. Cy, cytostome; K, kinetoplast. Bars = 1 μm.

SEM microphotographs of *T. ralphi* (Figure 6a,c,e,f,h,i) and *T. terena* (Figure 6d,g,j,k) show flagellates varying from small and rounded forms to long epimastigotes. The epimastigotes typically divide by binary fission (Figure 6j) while rounded flagellates divided by multiple fission as squash-like forms (Figure 6c,g). Epimastigotes of *T. terena* displayed a surface protuberance correspondent to the subjacent kinetoplast (Figure 6k). Both trypanosome species exhibited a cytostome opening, represented by a small orifice in the cell membrane close to the flagellar pocket (Figure 6f). In addition to areas of detachment of the flagellum and the cell body membranes (Figure 6d,h,j,k), the two species exhibited highly developed flagellar lamellae (Figure 6i,j).

**Figure 7:**
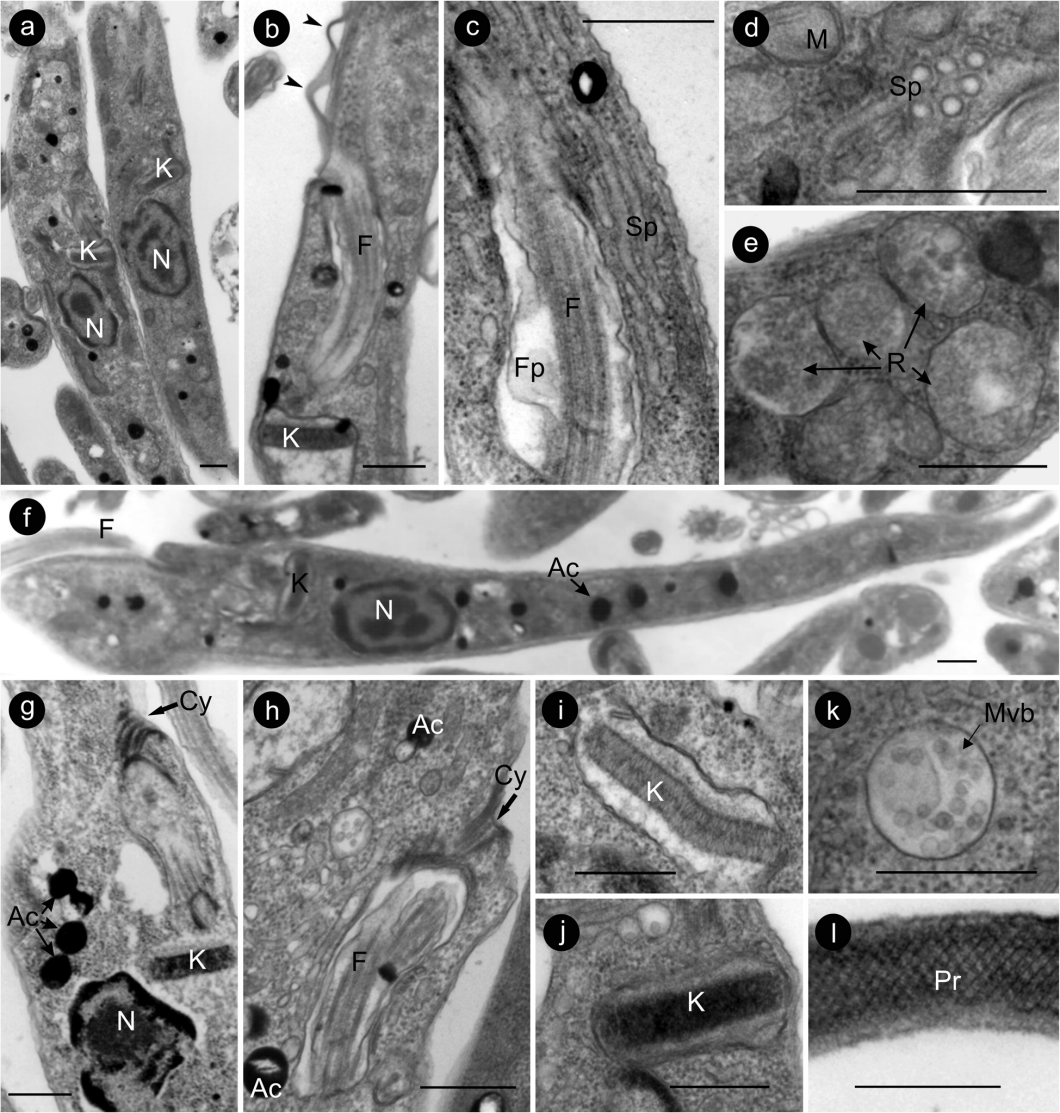
**Transmission electron microscopy showing the ultrastructural organisation of *Trypanosoma ralphi *(a,b,c,e,h,i) and *Trypanosoma terena *(d,f,g,j,k,l)*.*** Longitudinal sections of log-phase flagellates showing elongated epimastigotes **(a-c).** Network of microtubules near to flagellar pocket forming the espongiome in longitudinal **(c)** and transversal **(d)** sections. Both species exhibited cytostome **(g,h)**, multiple acidocalcisomes **(f,g,h)**, organelles resembling reservosomes **(e)**, multivesicular bodies **(k)** and a well-developed paraflagelar rod **(l)**. The arrangement of the kDNA fibrils in *T. ralphi* produced a two-band pattern **(i)** differing from the more compacted kDNA of *T. terena***(j)**. Arrowheads **(b)** indicate regions where the flagellar membrane detached from the cytoplasmatic membrane. N, nucleus; M, mitochondrion; K, kinetoplast, F, flagellum; Fp, flagellar pocket; Cy, cytostome; Sp, spongiome; Pr, paraflagellar rod; Ac, acidocalcisomes; R, reservosome; Mvb, multivesicular body. Bars = 0.5 μm.

The ultrastructural organisation of *T. terena* and *T. ralphi* were not particularly distinct from one another or from other trypanosomatids [14,19,37-39]. However, some features shared by both species were noticeable: a) large amount of organelles resembling reservosomes [19,39], which are compartments that accumulate endocytotic macromolecules (Figure 7e); b) an apparent short invagination of the cytostome inside the cytoplasm (Figure 7g,h); c) very well developed spongiomes [37], which are a network of tubules converging to the contractile vacuole attached to the membrane of the flagellar pocket constituting the osmoregulatory apparatus (Figure 7c,d), and d) a conspicuous paraflagelar rod (PR), a network of filaments that runs alongside the flagellar microtubules (Figure 7l). The thickness and the arrangement of the kDNA fibrils differed between the two species. In *T. ralphi* (Figure 7i), the kinetoplast exhibits a disk-shaped structure with two bands of fibrils and thickness varying from 170 to 190 nm (176.7± 6.5 nm). In *T. terena* (Figure 7j), the kinetoplast is highly compacted with no visible particular organization, and the thickness varied from 162 to 241 nm (198 ± 22 nm).

### Taxonomical summary

#### New species description

Phylum Euglenozoa Cavalier-Smith 1981; Class Kinetoplastea Honigberg 1963; Order Trypanosomatida (Kent 1880) Hollande 1982; Family Trypanosomatidae Doflein 1951; Genus *Trypanosoma* Gruby 1843; *Trypanosoma ralphi* Teixeira and Camargo n. sp. and *Trypanosoma terena* Teixeira and Camargo n. sp.

*Trypanosoma ralphi* Teixeira and Camargo n. sp.

#### Type material

Hapantotype, culture of the isolate TCC 1838; paratypes: TCC624, 625, 1092, 1100, 1101, 1119, 1120, 1829, 1974 and 2218. The hosts and collection locations of paratypes are in Table 1. Type host: *Melanosuchus niger* (Crocodylia, Alligatoridae). Additional hosts: *Caiman crocodilus* and *Ca. yacare.* Habitat*:* Blood. Locality: Purus river, Western Amazonas, Brazil (S7^o^15’ W64^o^47’). Additional collection localities: indicated in the Figure 1 and Table 1. Morphology: Log-phase epimastigotes averaging 23.26±4.48 μm long and 2.76±0.50 μm wide (Figure 5c). Diagnosis: The diagnosis was based on DNA sequences from the isolate TCC 1838 deposited in Genbank: SSU rRNA (KF546527) and gGAPDH (KF546512). Etymology: the name *T. ralphi* honours Dr. Ralph Lainson, a respected British protozoologist particularly known for his work on the leishmaniae, who lives in the Brazilian Amazônia enthusiastically studying haemosporidians of reptiles.

*Trypanosoma terena* Teixeira and Camargo n. sp.

#### Type material

Hapantotype, culture TCC 610. Type host: *Caiman yacare* (Alligatoridae, Crocodylia). Habitat*:* blood. Locality: Miranda River, State of Mato Grosso do Sul, Brazil (S20^o^14’ W56^o^22’). Because of the relevant genetic divergences, the isolates from *Ca. yacare*, *M. niger, Ca. crocodilus* and *O. tetrapsis* clustering with the isolate TCC 610 in the subclade *T. terena* were not designated paratypes of this species but merely subclade genotypes; their host species and collection sites are indicated in the Figure 1 and Table 1. Morphology: Log-phase epimastigotes averaged of 22.20±3.72 μm long and 2.06±0.44 μm wide (Figure 5g). Diagnosis: based on DNA sequences of the isolate TCC 610 deposited in GenBank: SSU rRNA (EU5962522) and gGAPDH (EU596256). Etymology: “*terena*”, name in apposition, refers to the indigenous Terena ethnic group inhabiting the region of the Pantanal where the first isolates of this species were obtained.

The cryopreserved cultures, Giemsa-stained smears and DNA samples from cultures and crocodilian blood samples were all deposited in the TCC-USP. To comply with the regulations of the International Code of Zoological Nomenclature (ICZN), details of the two species have been submitted to ZooBank with the following Life Science Identifier (LSID): urn:lsid:zoobank.org:pub:CF0B3CA9-C42A-4F76-B60F-DCD1771B79CA

#### Taxonomical comments

Lainson [20] described *Trypanosoma cecili* based on flagellates observed in tissue imprints of *Ca. crocodilus* captured at Eastern AM basin; cultures have never been obtained and no molecular data are available. The forms of *T. cecili* resemble those we previously described in lung imprints from *Ca. yacare*[21], but we cannot attest whether these forms correspond to one or more trypanosome species. We demonstrated that *Ca. crocodilus* captured in the AM, AT and OR basins can harbour at least three genetically different trypanosomes. Because the holotype of *T. cecili* is lost, no trypanosome could be elected as a neotype for *T. cecili*. Therefore, the trypanosomes herein analysed must be described as new species.

## Discussion

In this study, we assessed the genetic diversity and phylogenetic relationships of trypanosomes from South American alligatorids and African crocodilids, and compared phylo- and biogeographical patterns of these parasites with those of their host species to uncover possible events that could help to hypothesize evolutionary scenarios. The phylogenetic analyses enabled the description of *T. ralphi* n. sp and *T. terena* n. sp from Brazilian alligatorids. Data on culture behaviour, morphology and ultrastructural features complemented the species description.

The crocodilian trypanosomes from Africa and South America clustered together forming the strongly supported Crocodilian clade, which comprised three subclades led respectively by *T. terena*, *T. grayi* and *T. ralphi*. Unexpectedly, a trypanosome from the African *O. tetraspis* clustered tightly within the South American *T. terena* subclade instead of the *T. grayi* subclade exclusive of African trypanosomes*.* Also unpredictably, the *T. ralphi* subclade, which comprises exclusively South American isolates, was more closely related to the African *T. grayi* subclade than to the South American *T. terena* subclade.

The close genetic relationships between South American and African crocodilian trypanosomes is incompatible with the Cretaceous (~90 mya) split of Crocodylidae and Alligatoridae [28,30,32]. The results are more consistent with the paleontological and phylogenetic evidence of *Crocodylus* marine circumtropical dispersal at the Miocene/Pliocene boundary (~4-5 mya) [31-33]. Therefore, we hypothesised that marine dispersion played an important role in determining the close relationships between South American and African crocodilian trypanosomes. This scenario favours oceanic dispersion as the determinant of the disjunct distribution of crocodilian trypanosomes, that is, the discontinuous distribution of closely related trypanosomes of hosts separated by large geographical distances [40]. Disjunct distribution can also be demonstrated for other trypanosomes such as the homogeneous *T. lewisi* and *T. theileri,* which are distributed throughout the word, probably by the anthropogenic dispersal of their respective domestic rat and cattle hosts [11,16,18,39,41,42].

Our findings are in accordance with paleontological and phylogenetic evidence that *Crocodylus* species colonized the Neotropics and Afrotropics in the late Miocene (~5 mya) through transoceanic circumtropical dispersion. The dispersal of *Crocodylus* from Australasia proceeded to the Neotropics and then to Africa or inversely to Africa and then to the Neotropics [31-33]. Whatever the route, there was a profusion of alligatorids at the South American hydrographic basins when the ancestors of *Crocodylus* reached the Neotropics [33,35,36,43]. At that time, Amazonia was part of a much larger wetland extending from the OR to the PP basins without barriers separating the large and diverse crocodilian populations. The South American basins reached its present configuration at the Pliocene (~3 mya), following successive disconnections and reconnections due to marine and river drainage changes accompanying the Andean uplift. The present day basins are still interconnected although at a much smaller scale [43-46]. Resident alligatorids and the new arriving crocodilids shared wetlands and lakes where successive episodes of trypanosome host switches, apparently restricted to phylogenetically related hosts, may have occurred [14,16-18,47,48]. Host switching mediated by ecological fitting appear to be commonly used for trypanosome jumping among hosts that share ecological niches and can, therefore, serve as blood source for the same hematophagous vectors [12-16,19,47-50]. However, this part of the biogeographical history of crocodilian trypanosomes requires data about their transmission in South America. Tsetse flies, which cyclically transmit *T. grayi*, are widespread in Sub-Saharan Africa, and also occur in a few spots of the Arabian Peninsula. However, there is fossil evidence indicating tsetse dispersal into the Nearctic at the Miocene [51]. The virtual absence of tsetse flies in the location at Guinea Bissau where the trypanosome from *O. tetrapsis* was obtained suggests the existence of alternative vectors for crocodilian trypanosomes in Africa. In South America, different haematophagous vectors could transmit the crocodilian trypanosomes either cyclically or mechanically. We are currently searching for possible vectors among leeches and insects aiming to provide new insights into the evolutionary history of crocodilian trypanosomes.

Comparative phylogeography using parasites as biological tags has been valuable to track vertebrate hosts and vectors in time and space [1-5,18,39,52-54]. The finding that all crocodilian trypanosomes, whatever their vectors, clustered together forming a clade exclusively of crocodilian trypanosomes, regardless of host species and geographic origin, indicated that the evolution of these trypanosomes was strongly linked to that of their vertebrate hosts. Our phylogeographical analysis can contribute to the understanding of the worldwide dispersal of crocodilians, similarly to studies on bat trypanosomes that indicated recent movements of bats that could not be inferred from available paleontological and phylogenetic data [11,39,41]. Furthers studies including more comprehensive sampling of trypanosomes from crocodilians, in Africa, Asia and the Americas, and their vectors are required for an improved hypothesis about the origin, dispersal route and evolutionary relationships of crocodilian trypanosomes.

## Conclusions

Phylogenetic and paleontological data on crocodilians and phylogenetic and biogeographical data on their trypanosomes are consistent with past and present biogeographical scenarios playing important roles in the genetic diversity, and phylogenetic relationships of crocodilian trypanosomes. In the most likely scenario, the evolutionary history of crocodilian trypanosomes was shaped in response to the ancient geographical isolation between alligatorids and crocodilids, followed by the transcontinental voyage of *Crocodylus* at the Miocene allowing the contact between South American alligatorids and crocodilids. This contact probably enabled successive host switches of trypanosomes between resident alligatorids and migrating crocodiles throughout the configuration of the present day South American hydrographic basins.

## Competing interests

The author(s) declare that they have no competing interests.

## Authors’ contributions

MMGT and EPC conceived the study and wrote the manuscript and both are senior authors. FP, BRF, LBV, HAG, CDP and RBA designed and implement the sample collection and provided information to prepare the manuscript; MC and CSAT performed culturing and morphological analysis; BRF, LBV and PBH have done the phylogenetic analyses and provided suggestions to improve the manuscript. All authors read, revised, and approved the final version of the manuscript.
